# Deep-Subsurface Pressure Stimulates Metabolic Plasticity in Shale-Colonizing *Halanaerobium* spp.

**DOI:** 10.1128/AEM.00018-19

**Published:** 2019-05-30

**Authors:** Anne E. Booker, David W. Hoyt, Tea Meulia, Elizabeth Eder, Carrie D. Nicora, Samuel O. Purvine, Rebecca A. Daly, Joseph D. Moore, Kenneth Wunch, Susan M. Pfiffner, Mary S. Lipton, Paula J. Mouser, Kelly C. Wrighton, Michael J. Wilkins

**Affiliations:** a Department of Microbiology, Ohio State University, Columbus, Ohio, USA; b Environmental Molecular Sciences Laboratory, Pacific Northwest National Laboratory, Richland, Washington, USA; c College of Food, Agricultural, and Environmental Sciences, Ohio State University, Columbus, Ohio, USA; d Biological Sciences Division, Pacific Northwest National Laboratory, Richland, Washington, USA; e DowDuPont Industrial Biosciences, Wilmington, Delaware, USA; f Center for Environmental Biotechnology, University of Tennessee, Knoxville, Tennessee, USA; g Department of Civil and Environmental Engineering, University of New Hampshire, Durham, New Hampshire, USA; h Department of Soil and Crop Sciences, Colorado State University, Fort Collins, Colorado, USA; Chinese Academy of Sciences

**Keywords:** *Halanaerobium*, shale, biofilms, high pressure, hydraulic fracturing, metabolomics

## Abstract

Bacterial *Halanaerobium* strains become the dominant persisting microbial community member in produced fluids across geographically distinct hydraulically fractured shales. *Halanaerobium* is believed to be inadvertently introduced into this environment during the drilling and fracturing process and must therefore tolerate large changes in pressure, temperature, and salinity. Here, we used a *Halanaerobium* strain isolated from a natural gas well in the Utica Point Pleasant formation to investigate metabolic and physiological responses to growth under high-pressure subsurface conditions. Laboratory incubations confirmed the ability of Halanaerobium congolense strain WG8 to grow under pressures representative of deep shale formations (21 to 48 MPa). Under these conditions, broad metabolic and physiological shifts were identified, including higher abundances of proteins associated with the production of extracellular polymeric substances. Confocal laser scanning microscopy indicated that extracellular polymeric substance (EPS) production was associated with greater cell aggregation when biomass was cultured at high pressure. Changes in *Halanaerobium* central carbon metabolism under the same conditions were inferred from nuclear magnetic resonance (NMR) and gas chromatography measurements, revealing large per-cell increases in production of ethanol, acetate, and propanol and cessation of hydrogen production. These metabolic shifts were associated with carbon flux through 1,2-propanediol in response to slower fluxes of carbon through stage 3 of glycolysis. Together, these results reveal the potential for bioclogging and corrosion (via organic acid fermentation products) associated with persistent *Halanaerobium* growth in deep, hydraulically fractured shale ecosystems, and offer new insights into cellular mechanisms that enable these strains to dominate deep-shale microbiomes.

**IMPORTANCE** The hydraulic fracturing of deep-shale formations for hydrocarbon recovery accounts for approximately 60% of U.S. natural gas production. Microbial activity associated with this process is generally considered deleterious due to issues associated with sulfide production, microbially induced corrosion, and bioclogging in the subsurface. Here we demonstrate that a representative *Halanaerobium* species, frequently the dominant microbial taxon in hydraulically fractured shales, responds to pressures characteristic of the deep subsurface by shifting its metabolism to generate more corrosive organic acids and produce more polymeric substances that cause “clumping” of biomass. While the potential for increased corrosion of steel infrastructure and clogging of pores and fractures in the subsurface may significantly impact hydrocarbon recovery, these data also offer new insights for microbial control in these ecosystems.

## Abstract (precis)

The hydraulic fracturing of deep-shale formations for hydrocarbon recovery accounts for approximately 60% of U.S. natural gas production. Microbial activity associated with this process is generally considered deleterious due to issues associated with sulfide production, microbially induced corrosion, and bioclogging in the subsurface. Here we demonstrate that a representative *Halanaerobium* species, frequently the dominant microbial taxon in hydraulically fractured shales, responds to pressures characteristic of the deep subsurface by shifting its metabolism to generate more corrosive organic acids and produce more polymeric substances that cause “clumping” of biomass. While the potential for increased corrosion of steel infrastructure and clogging of pores and fractures in the subsurface may significantly impact hydrocarbon recovery, these data also offer new insights for microbial control in these ecosystems.

## INTRODUCTION

The hydraulic fracturing (HF) of subsurface formations to release economically important hydrocarbons generates extensive fracture networks in these deep-subsurface ecosystems. Shales are thought to be almost sterile prior to HF due to a range of factors, including prior “paleo-pasteurization” coupled with extremely low levels of permeability and nanometer-size pores that physically preclude the development of microbial ecosystems (1). However, microorganisms present in fluids that are injected into newly developed fracture networks during HF are able to colonize the system and persist over extended periods of time (2–4). Thus, microorganisms existing under ambient surface conditions are suddenly exposed to (and must respond to) dramatically different physicochemical conditions in deep-shale fracture networks characterized by anoxia, high temperatures, increasing salinity, and elevated pressures.

Prior work by our research group and others has demonstrated that microorganisms associated with the genus *Halanaerobium* are dominant persisting members of shale communities across geographically distinct shale plays (2–6). *Halanaerobium* spp. are frequently low-abundance community members (∼1%) in fracturing fluids, but they outcompete other taxa to become enriched in later-produced fluid samples (2). These Gram-positive microorganisms have also been observed in other saline environments, including conventional oil and gas reservoirs, and are able to grow on a range of carbon substrates, including sugars and guar gum (7). Importantly, these microorganisms occupy key roles in inferred metabolic networks that sustain microbial life in shale ecosystems, centered on the cycling of osmoprotectants and methylamine compounds (2, 8). The growth of such microorganisms in fractured shales is commonly viewed as deleterious, due to studies indicating that *Halanaerobium* spp. are able to catalyze thiosulfate-dependent sulfidogenesis (5), grow on additive chemicals present in input fluids (2, 7, 8), and potentially form biofilms in the subsurface. These processes could directly contribute to biofouling in the fracture network, leading to significant decreases in reservoir permeability and associated hydrocarbon recovery. While such processes are undesirable where hydrocarbons are being extracted, reductions in permeability in other geologic systems (e.g., sealing cap rock in geologic CO_2_ sequestration reservoirs) may be beneficial (9).

Studies have shown that the salinity of fluids within the shale fracture network rapidly increases over the first ∼75 days following the HF process, due to the dissolution of solid-phase salt minerals in the deep subsurface (8). Microorganisms in the fracture networks may protect themselves against high-salinity conditions through the import of ions (e.g., K^+^) or the utilization of osmoprotectant compounds (e.g., glycine betaine) that maintain intracellular cell turgor pressure (2, 10). While the mechanisms of microbial tolerance to high pressure are less well understood, recent experiments have indicated that utilization of osmoprotectants and intracellular tolerance to high salt concentrations may stabilize protein structure due to increases in hydrophobic interactions and reduced water activity (11, 12).

Here, we used a Halanaerobium congolense strain (WG8) isolated from produced waters from a hydraulically fractured well in the Utica Point Pleasant formation, Ohio, USA, to investigate cellular responses to high-pressure conditions characteristic of the deep terrestrial subsurface. Using high-pressure growth reactors, shotgun proteomic measurements and proton nuclear magnetic resonance (^1^H-NMR) metabolomics analyses, we identified the potential for increased production of extracellular polymeric substances (EPS) and altered central metabolism under pressurized growth conditions. Given that these changes resulted in increasing cell clumping and production of potentially corrosive organic acids, the results have implications for maintenance of fracture permeability and well integrity in unconventional systems in the presence of active microbial populations.

## RESULTS

### *Halanaerobium* growth under pressurized conditions.

**FIG 1:**
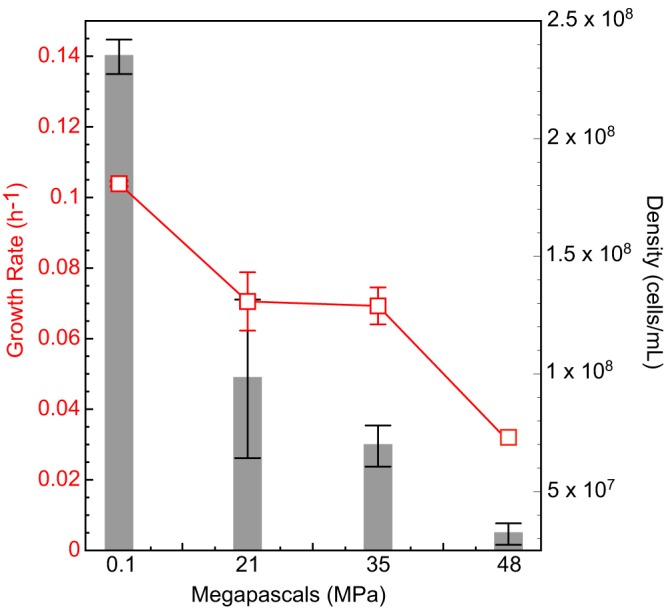
H. congolense WG8 growth at high pressure. Growth rates (red) and corresponding cell densities (gray) across a pressure gradient (0.1 to 48 MPa) are shown. Growth rate and cell densities decreased as incubation pressure increased.

Halanaerobium congolense WG8 was able to grow under both atmospheric pressure (0.1 MPa) and elevated pressure characteristic of deep-subsurface shales (21 to 48 MPa). Both the highest growth rate (0.104 h^−1^), and the highest biomass yield were measured under atmospheric-pressure incubation conditions, with growth rate and biomass yield decreasing with increasing pressure (growth rate = 0.071, 0.070, and 0.030 h^−1^ at 21, 35, and 48 MPa, respectively) (Fig. 1).

### Fermentation product profiles change under pressurized growth conditions.

**FIG 2:**
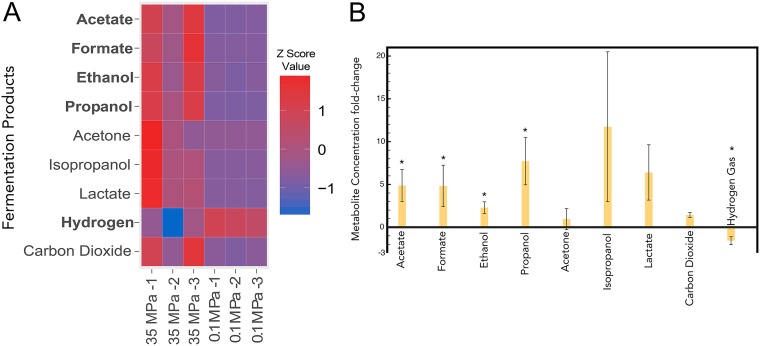
Major fermentation products excreted by H. congolense WG8 detected using NMR and gas chromatography. (A) The heat map shows the Z score (the number of standard deviations away from the mean) of normalized concentrations. Bolded product names signify differences in concentrations between treatments where *P* < 0.05. (B) Fold changes in per-cell fermentation products. All positive values represent increased production under high-pressure growth conditions. Asterisks represent statistically significant changes in fermentation product formation (*P* < 0.05).

In the deep-shale environment, it is hypothesized that the degradation and fermentation of chemical additives, such as guar gum, support at least some *Halanaerobium* growth (7). Given that this substrate is metabolized through central glycolysis, we provided glucose as a representative carbon source in the experiments described here. Proton nuclear magnetic resonance (^1^H-NMR) and gas chromatography (GC) were used to analyze *Halanaerobium* glucose fermentation product profiles following growth at atmospheric pressure (0.1 MPa) and 35 MPa. From these data, a fermentation balance using the oxidized and reduced products yielded a balanced ratio, indicating that all the major fermentation products were accounted for (see Table S1 in the supplemental material). Across the two growth conditions, acetate, formate, ethanol, propanol, acetone, isopropanol, lactate, hydrogen gas, and carbon dioxide were all identified as excreted fermentation products. Due to differences in cell yields under different growth conditions, the per-cell concentration of each product was calculated by dividing the concentration of each fermentation product by the cell density. Although the major fermentation products excreted under both pressure conditions were acetate, ethanol, and formate, pressurized growth led to increases in the per-cell concentration of all aqueous fermentative compounds (Fig. 2). Coupled to this increase in aqueous fermentation products, there was a concomitant 2-fold (30%) decrease in per-cell evolved hydrogen concentrations at 35 MPa. Low concentrations of propionate, propanol, isopropanol, and lactate that were identified under atmospheric pressure also showed differential changes in cultures grown at 35 MPa. Although propionate was no longer detected at 35 MPa, per-cell concentrations of propanol, isopropanol, and lactate increased 7-, 10-, and 5-fold, respectively (Fig. 2).

From proteomic data collected at 0.1, 21, 35, and 48 MPa, we infer that H. congolense WG8 uses the Embden-Meyerhof-Parnas pathway for pyruvate synthesis, with the major fermentation products (lactate, formate, ethanol, acetate, and CO_2_) generated via the mixed-acid fermentation pathway. While lactate dehydrogenase (EC 1.1.1.27; WG8-102157), pyruvate formate lyase (EC 2.3.1.54; WG8-1397), acetate kinase (WG8-10940 and -101139), and alcohol dehydrogenase (WG8-10941, -11552, -11320, -11934, -1491, and -1513) were identified across all four pressure incubation conditions, no close match to formate dehydrogenase or formate:hydrogen lyase was found in either the H. congolense WG8 proteomic data set or the genome sequence. Instead, we infer that activity of a pyruvate-ferredoxin oxidoreductase (WG8-11648) is responsible for the similar per-cell CO_2_ concentrations detected using gas chromatography at both 0.1 and 35 MPa (13). Pyruvate-ferredoxin oxidoreductase uses pyruvate, coenzyme A (CoA), and oxidized ferredoxin to produce acetyl-CoA, CO_2_, reduced ferredoxin, and H^+^, and it was detected in the proteomic data across all four pressure growth conditions. The decrease in per-cell H_2_ production at 35 MPa growth conditions was coupled with a decrease in hydrogenase protein abundances at increased pressures (21 to 48 MPa) (see Table S2 and Fig. S1 in the supplemental material).

### Global proteome profiles indicate shifts in *Halanaerobium* physiology and metabolism at high pressure.

Label-free shotgun proteomic analyses were subsequently used to infer metabolic and physiological changes associated with the observed *Halanaerobium* growth and metabolite profiles across all four growth conditions. There are 2,547 predicted protein-coding genes within the H. congolense WG8 genome, and 1,826 of these proteins were identified within the proteomic data set. Only a subset of 255 proteins were found at statistically significant higher abundances when *Halanaerobium* was grown under pressure (Student’s *t* test, *P* < 0.05 in two of three high-pressure conditions). Of these 255 proteins, 77 were identified only under high-pressure growth conditions and 85 were present in higher abundance under all three pressurized growth conditions.

Decreasing H. congolense WG8 growth rates and cell yields (Fig. 1) suggested that these cultures were stressed when incubated under high-pressure conditions. No novel bacterial growth mechanism for high-pressure survival has been established, but other high-pressure studies have identified similar stress-induced proteins expressed across many environmental stresses, such as temperature, salt, and pH (14). Supporting this inference, proteins associated with diverse stress responses were present solely when strain WG8 was grown at high pressure, including a universal stress response protein (UspA; WG8-10868) and enzymes that regulate intracellular redox conditions (thioredoxin; WG8-12911). Other proteins, including alkaline shock proteins (WG8-1014 and -1015) and heat shock proteins (WG8-1189, -11129, and -10522) were measured under all conditions but at higher abundances under high pressure. Heat shock proteins have been associated with high-pressure growth in Escherichia coli and act to maintain the native composition of proteins, making them indicative of piezotolerant organisms responding to increased pressure (14, 15). Additionally, alkaline shock proteins have been shown to be more abundant in Staphylococcus aureus biofilms than in planktonic cells (16). F-type ATPases (WG8-10748, -10751, -10752, and -10753) were also present at higher abundances in biomass incubated at high pressure and are believed to aid in high-pressure adaption by maintaining the cellular energy supply when under stress (14). Lastly, deep-sea piezophiles accumulate osmolytes that help protect against oxidants (e.g., free radicals) that are generated under stresses such as high pressure or salinity (14). *Halanaerobium* WG8 utilizes osmolytes when it is grown at low and high pressure, with a sodium/hydrogen antiporter detected under both growth conditions (WG8-10764, -11350, and -101143). Certain amino acids such as glutamate and glycine may also act as osmolytes, and proteins involved in their synthesis were in higher abundance when *Halanaerobium* WG8 was grown at high pressure (14). These proteins include glutamate synthase (WG8-11426), glycine hydroxymethyltransferase (WG8-105103), and glycine dehydrogenase (WG8-1167).

**FIG 3:**
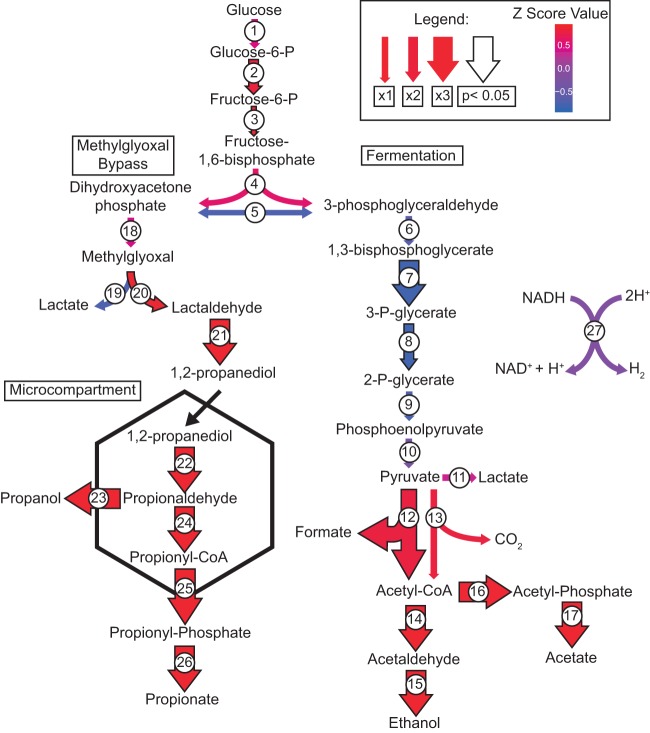
Predicted carbon flux through H. congolense WG8 when grown under pressure (21, 35, and 48 MPa) using proteomic and NMR analyses. H. congolense WG8 is a strict fermenter, and glucose was the substrate provided during growth experiments. Major fermentation products are acetate, ethanol, formate, lactate, propanol, carbon dioxide, and hydrogen gas (only when grown at 0.1 MPa). The production of 1,2 propanediol is hypothesized to be a result of the methylglyoxal bypass, which may become important during high-pressure growth because activity of triose phosphate isomerase (protein 5) decreases under pressure. 1,2-Propanediol and other alcohols converted into aldehydes are processed in a microcompartment to contain toxic aldehyde intermediates. The arrow size represents the increased abundance of a protein under 1, 2, or 3 high-pressure growth conditions. Arrows outlined in black represent statistically significant changes in protein abundance (*P* < 0.05). Arrow colors are based on Z-score values calculated from protein abundances. Proteins: 1, phosphotransferase; 2, glucose-6-phosphate isomerase; 3, phosphofructokinase; 4, fructose bisphosphate aldolase; 5, triose phosphate isomerase; 6, glyceraldehyde-3-phosphate dehydrogenase; 7, 3-phosphoglycerate kinase; 8, phosphoglycerate mutase; 9, enolase; 10, pyruvate kinase; 11, lactate dehydrogenase; 12, pyruvate formate lyase; 13, pyruvate-ferredoxin oxidoreductase; 14, aldehyde dehydrogenase; 15, alcohol dehydrogenase; 16, phosphotransacetylase; 17, acetate kinase. Methyl glyoxal bypass: 18, methylglyoxal synthase; 19, glyoxalase; 20, methylglyoxal reductase; 21, 1,2-propanediol dehydrogenase. Microcompartment: 22, propanediol dehydratase; 23, alcohol dehydrogenase; 24, propionaldehyde dehydrogenase; 25, phosphotransacetylase; 26, propionate kinase; 27, hydrogenase.

Proteomic data analyses revealed a strong signal for the utilization of 1,2-propanediol by H. congolense WG8 under high-pressure growth conditions. In model organisms (e.g., *Salmonella*) this compound is used as a carbon substrate via the formation of propionyl-CoA that eventually feeds into the tricarboxylic acid (TCA) cycle as pyruvate (17). The utilization of 1,2-propanediol typically occurs in intracellular compartments known as carboxysomes or bacterial microcompartments. In H. congolense WG8, the genes for 1,2-propanediol utilization and microcompartment synthesis are present in a single operon (WG8-10936 to -10958). All proteins encoded by these genes were both detected and present at higher abundances under pressurized growth conditions (Fig. 3). These included all three subunits of the propanediol dehydratase (PduCDE; WG8-10954 to -0956), which catalyzes the formation of propionaldehyde from 1,2-propanediol, and a propionaldehyde dehydrogenase (PduP; WG8-10944) that converts propionaldehyde to propionyl-CoA. Seven proteins involved in microcompartment generation were additionally more abundant at pressure and are likely play a critical role in protecting H. congolense WG8 from intracellular toxicity associated with propionaldehyde formation.

Cofactor B_12_ (adenosylcobalamin) is required by propanediol dehydratase for the aforementioned conversion of 1,2-propanediol to propionaldehyde. Consequently, all proteins involved in uroporphyrinogen synthesis (WG8-10961 to -10963) and the conversion of precorrin to adenosylcobalamin were present at higher abundances in high-pressure samples (Fig. 3 and S1). Reflecting the presence of a cobalt active site in adenosylcobalamin, three cobalt transporters (WG8-10976, -10979, and -11250) were present at higher abundances in pressure-grown cells, as were outer membrane TonB-dependent transporters (WG8-102136, -10244, -10246, -10245, -102135, and -102134) involved in B_12_ and iron intracellular transport.

**FIG 4:**
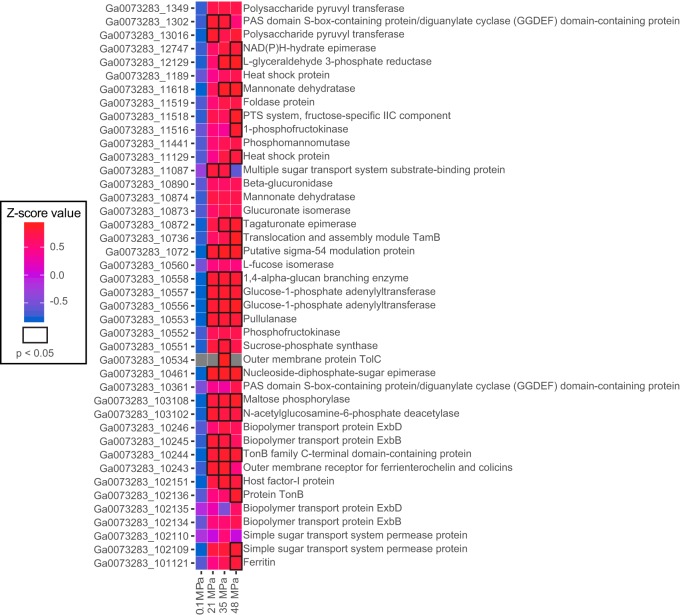
Proteins potentially involved in stress response and associated EPS formation in H. congolense WG8. Black outlined boxes represent a significant difference (*P* < 0.05, Student’s *t* test) in protein abundances between low- and high-pressure conditions. Boxes without outlines represent changes in protein concentration that were not statistically significant (*P* > 0.05, Student’s *t* test).

**FIG 5:**
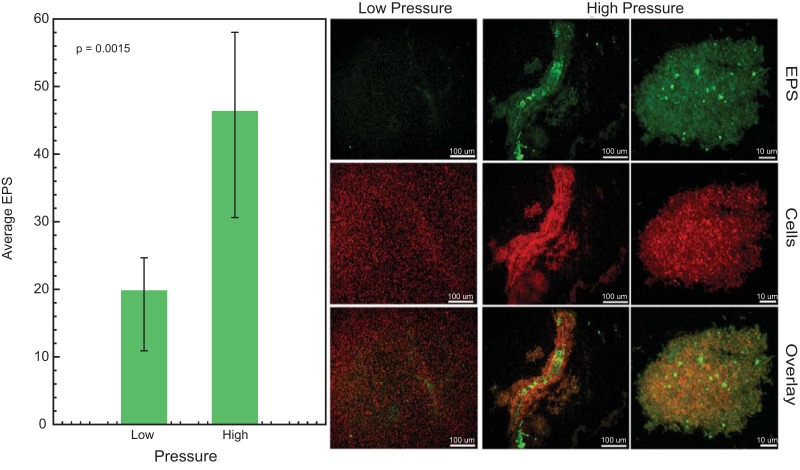
Confocal scanning laser microscopy analysis of *Halanaerobium* grown at high and low pressure. The bar graph represents the average amount of extracellular polymeric substance produced by the cells in three biological replicates grown at high and low pressure (*P* < 0.05, Student’s *t* test). The confocal image panel shows examples of *Halanaerobium* biofilms. Syto59 (red) was used to stain nucleic acids, while Alexa Fluor 488-ConA (green) was used to stain α-mannopyranosyl and α-glucopyranosyl residues within the EPS matrix.

Cell clumping was observed when *Halanaerobium* biomass was incubated under pressurized conditions. Approximately 39 proteins previously implicated in biofilm formation and extracellular polymeric substance (EPS) synthesis in other microorganisms were present at higher abundances in cell cultures grown at high pressure (Fig. 4) (18–29). These proteins were associated with membrane transport (TonB [30] [WG8-10244 and -102136] and ferritin [31] [WG8-101121 and -10243]), sugar biosynthesis (epimerases [32] [WG8-10872] and isomerases [33] [WG8-10873]), sugar transport (TamB [34] [WG8-10736] and TolC [35] [WG8-10534]), and glycogen formation (pullulanase [36] [WG8-10553]) and could contribute to increase EPS production and surface attachment under pressurized growth (Fig. 5). The potential role of cyclic di-GMP in stimulating biofilm formation was inferred by the presence of three diguanylate cyclase domain-containing proteins (37) (WG8-11421, -1302, and -10361), two of which were present solely in cells from high-pressure incubations (Fig. 4).

### *Halanaerobium* exhibits cell clumping behavior at high pressure.

To quantify EPS formation and cell clumping by *Halanaerobium*, cultures incubated under atmospheric and high-pressure conditions were imaged using confocal laser scanning microscopy (CLSM). Cells incubated at 35 MPa generated approximately 6 times more EPS than those incubated at 0.1 MPa (Fig. 5). Floating clusters of biomass were more common when *Halanaerobium* was grown at high pressure (Fig. 5).

### PFLA acid profiles change across pressure incubation conditions.

Phospholipid fatty acid (PLFA) data were obtained from silicic acid chromatography via esterification and gas chromatography-mass spectrometry (GC-MS) for cultures grown under atmospheric (0.1 MPa) to high (up to 48 MPa) pressures. PLFA profiles were compared based on both the relative abundance of identified biomarkers and normalization to cell density (pmol/cell). Cells grown under elevated pressures showed distinct changes in the abundance and structure of identified fatty acids. As pressure increased from atmospheric to 35 MPa, the relative abundance of both saturated and monosaturated PLFAs increased. However, during growth at the highest pressure (48 MPa), the relative abundance of saturated PLFAs (30%) was similar to that in cells grown under a surface atmosphere. Despite this similar abundance of saturated PLFAs at the highest and lowest pressures and a relatively even weighted chain length across all pressures (15.9 to 16.3), *Halanaerobium* cells grown at 48 MPa reduced their synthesis of monounsaturated PLFAs by more than half (14.6%) relative to cells grown at atmospheric pressure (35.2%) (see Table S3 in the supplemental material). PLFAs absent at high pressure included iso- and anteiso-monounsaturated fatty acids (iso-C_15:1ω5t_ and anteiso-C_15:1ω5t_) as well as C_14:1ω5c_ and C_18:1ω9t_. This decrease in the degree of unsaturation at higher pressure has been observed in Bacillus cereus isolated from a deep-sea environment in cultures grown under anaerobic, low-temperature conditions. (38, 39). A more saturated phospholipid bilayer allows for tighter packing of the membrane, increased thickness of the lipid bilayer, and a decrease in membrane fluidity for higher rigidity (38–42). We also observed an increase in three monounsaturated fatty acids (C_18:1ω9c-ep_, C_18:1ω9t-ep_, and C_18:1-OH9,10_), the saturated palmitic acid (C_16:0_), one oxirane (C_18:0-OX9_), and two cyclopropanes (C_17:0Δ 9,10c_ and C_17:0Δ 9,10t_) for cells grown under pressure. An increased degree of cyclization and increased straight-chain fatty acid composition are associated with membrane bulking and decreased membrane permeability (41, 43), adaptations which could be important for membrane integrity under elevated pressure (42). Indeed, increasing amounts of monounsaturated C_18:1_ fatty acids under pressurized growth conditions have previously been reported in the piezophilic deep-sea bacterium Photobacterium profundum SS9, where they are thought to create local regions of fluidity around membrane-bound proteins that prevent the rigidity from inhibiting their activity (41). While a similar study in another model bacterium, Shewanella piezotolerans WP3, identified monounsaturated fatty acids, their role in high-pressure adaptation was less clear (42). In conclusion, we infer that although the lipid bilayer as a whole becomes more rigid to decrease permeability at high pressure, regionalized pockets of fluidity are maintained via increases in monounsaturated fatty acids to allow continued function of membrane proteins.

## DISCUSSION

*Halanaerobium* is detected as a dominant microbial community member across geographically distinct deep fractured shale ecosystems (8). Other studies have shown that the *Halanaerobium* relative abundance increases within hydraulically fractured shale microbial communities as salinity increases above 10% total dissolved solutes (2). While *Halanaerobium* is able to grow across a broad salinity range, its metabolic flexibility also likely plays a key role in its ability to colonize and persist within these ecosystems. Here the effects of subsurface pressure on the growth rate of Halanaerobium congolense WG8 were calculated from laboratory incubations, and the results suggest that H. congolense WG8 is piezotolerant rather than piezophilic (44). We hypothesize that while this microorganism may effectively grow in a currently unidentified surface ecosystem associated with the hydraulic fracturing process (e.g., water tanks or drill muds), it is also able to grow at pressures characteristic of the deep subsurface, albeit at lower rates.

Metabolite profiles suggest that pressurized growth was associated with broad-scale changes in central metabolism and production of fermentation end products. Under atmospheric pressure, H. congolense WG8 disposes of reductant via the generation of gaseous (H_2_) and aqueous (ethanol, acetate, and formate) fermentation products. Under high pressure, H. congolense WG8 reduced the per-cell generation of H_2_ but increased the production of lactate and alcohols as a mechanism for continued removal of reducing equivalents. While additional small shifts in metabolite profiles could be attributed to pressure-induced pH changes, we anticipated that the pH in the buffered growth medium would not vary significantly under the different pressure growth conditions. The oxidation/reduction potentials of these fermentation products were successfully balanced for high (35 MPa)- and low (0.1 MPa)-pressure growth (1.14 and 0.91, respectively), indicating that the production of increased lactate and alcohols compensated for the loss of gaseous H_2_ (Fig. 2; see Table S2 in the supplemental material). Hydrogenases act as electron sinks for fermentative organisms, and their inactivity leads to an increased electron pool available for alcohol and organic acid production (45). Higher alcohol production has been demonstrated in fermenting Clostridium thermocellum mutants with inactivated hydrogenases (45), while lower activity of hydrogenases under supraoptimal pressurized growth conditions has previously been observed in the piezophilic microorganism Pyrococcus yayanosii CH1 (46). While the exact physiological or metabolic driver for these trends is not completely understood, it has been suggested that decreasing abundances of hydrogenase enzymes and associated H_2_ production may be associated with adaptation of cell membrane-embedded proteins to changing membrane fluidity (46, 47). The increased production of potentially corrosive organic acids in response to decreasing hydrogenase activity may have implications for steel infrastructure in the subsurface. Acetate can drive corrosion of carbon steel in high-salinity environments (48), and therefore, metabolic shifts that favor organic acid production under high pressure may represent another potential issue associated with persistence of fermentative microorganisms such as *Halanaerobium* in fractured shale networks.

Exposure to high-pressure conditions characteristic of deep-shale ecosystems induced a strong proteomic signal for 1,2-propanediol processing, despite the addition of glucose as the sole carbon substrate in culture media. During glycolysis, fructose bisphosphate is converted to both dihydroxyacetone phosphate (DHAP) and glyceraldehyde 3-phosphate (G3P). Under atmospheric pressure, the activity of triose phosphate isomerase immediately converts DHAP to G3P, which is subsequently processed through stage 3 of glycolysis to pyruvate. However, elevated pressures have been shown to reduce the activity of glyceraldehyde 3-phosphate dehydrogenase, which converts G3P to 1,3-bisphosphoglycerate (49). Under such conditions, DHAP formation from fructose bisphosphate is favored, and it is potentially processed to d-lactate and 1,2-propanediol through the methylglyoxal bypass (Fig. 3). All proteins required for the methylglyoxal bypass (methylglyoxal synthase, methylglyoxal reductase, 1,2-propanediol dehydrogenase, and glyoxalase) were observed in proteomic data sets and were present at higher abundances under pressure.

In H. congolense WG8, we hypothesize that the methylglyoxal bypass is used for the removal of DHAP and the disposal of reducing equivalents through oxidation of NADH to NAD^+^ via methylglyoxal reductase and 1,2-propanediol dehydrogenase activity. The removal of reductant in this pathway may be important given the inferred decreases in activity of hydrogenase enzymes during high-pressure growth. Additional removal of DHAP may occur through the conversion of DHAP to dihydroxyacetone via dihydroxyacetone kinase (39). Dihydroxyacetone have been shown to accumulate in deep-sea microbial communities and is believed to aid in high-pressure adaption (39). While dihydroxyacetone was not directly measured in this study, dihydroxyacetone kinase was more abundant at high pressure and could have utilized some of the DHAP pool. For DHAP incorporated into the methylglyoxal bypass, the resulting 1,2-propanediol is shuttled through the propanediol utilization pathway, consisting of 21 proteins that were all present at statistically significant higher abundances when H. congolense WG8 was grown under high pressure. This pathway converts 1,2-propanediol to propanol and propionate, but key intermediates are propionaldehyde and propionyl-CoA. The formation of propionaldehyde and propionyl-CoA takes place within a synthesized microcompartment, due to both the toxicity of propionaldehyde and the requirement for close proximity between cofactor B_12_ and the propanediol dehydratase active site (50). The microcompartment also protects the radical intermediate formed in the active site of diol dehydratase from escaping or being quenched by undesirable side reactions, which would make the enzyme permanently inactive (51, 52). The diol dehydratase reactivation enzymes, cobalamin reductase, and adenosyltransferase (WG8-10952/10953, -10943, and -10945) reactivate the dehydratase active site via replacement of the cofactor B_12_ molecules.

The presence of propanol at higher per-cell concentrations under high-pressure growth conditions provides additional evidence for the activity of this pathway in H. congolense WG8. The cell is able to dispose of additional reducing equivalents via the oxidation of NADH to NAD^+^ coupled to the conversion of propionaldehyde to 1-propanol. If propionaldehyde is instead converted to propionyl-CoA, the precursor to propionate, NADH is generated (Fig. 3). We hypothesize that the requirement to recycle reducing equivalents favors formation of 1-propanol over propionate and can account for the near absence of propionate in the extracellular medium under high-pressure growth conditions.

**FIG 6:**
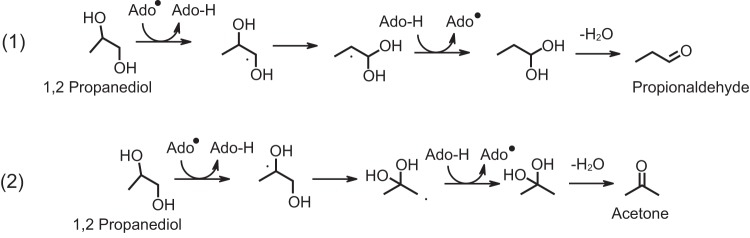
Proposed mechanism for diol dehydratase-catalyzed reactions. This mechanism involved a free radical-induced rearrangement of -OH groups to generate aldehydes and ketones. Reaction 1 is believed to be the most common route to generate propionaldehyde from 1,2-propanediol. Propionaldehyde is converted to 1-propanol. Reaction 2 could be induced under high-pressure conditions, leading to the formation of acetone from 1,2-propanediol. Acetone may be an isopropanol precursor. Ado^•^ denotes the 5′-deoxyadenosyl radical supplied by the coenzyme B_12_.

Other excreted fermentation products (isopropanol, ethanol, and acetate) were also present at higher per-cell concentrations under high-pressure growth conditions. The most-studied pathway for fermentative isopropanol formation is the isopropanol-butanol-ethanol pathway, which requires the enzymes acetoacetyl-CoA:acetate/butyrate:CoA transferase and acetoacetate decarboxylase, neither of which is present in the H. congolense WG8 genome (53). We hypothesize that isopropanol may instead be synthesized through the 1,2-propanediol utilization pathway, via acetone or propionaldehyde intermediates. Such a reaction requires rearrangement of either alcohol on the terminal and middle carbons in 1,2-propanediol (52); while alcohol rearrangement to the terminal carbon resulting in propanol formation is the most common route, high-pressure conditions could alter this process to generate increased concentrations of isopropanol (Fig. 6).

In addition to 1,2-propanediol, diol dehydratase and associated proteins can act on other 1,2-diols such as ethylene glycol and 2,3-butanediol, forming acetate and ethanol (52, 54, 55). Ethylene glycol is frequently present in chemical additives used in the hydraulic fracturing process, where it serves a multifunctional purpose as a cross-linker, friction reducer, gelling agent, and nonemulsifier. The activity of the diol dehydratase and associated proteins within *Halanaerobium* microcompartments suggests that these chemical additives could be degraded by microbial activity, with implications for the effectiveness of the compounds added into the shale formation.

High-pressure cultivation also induced a series of putative stress responses in H. congolense WG8. Previous studies have shown that antioxidant enzymes catalase, DNA-binding protein (Dps), alkyl hydroperoxide reductase, and DNA recombination protein RecA offer protection to the deep-sea bacterial strain Shewanella piezotolerans WP3 against oxidative stress induced by high pressure (56). Proteomic evidence revealed increased abundances of alkyl hydroperoxide reductase and DNA recombination protein RecA in *Halanaerobium* WG8 cultures grown at the highest-pressure treatment of 48 MPa. These proteins could be acting to defend the cell against oxidative stress induced by high pressure through signaling and DNA repair (56). Both heat and alkaline shock proteins that were more abundant in high-pressure incubations have been detected in E. coli cultured under similar conditions and are indicative of organisms responding to pressure and oxidative stress (47, 56). These proteins are believed to stabilize protein quaternary structure, thus maintaining membrane integrity, translation processes, and stability of macromolecules at high pressure (47). Under the same conditions, we also identified greater abundances of multiple proteins associated with EPS production and biofilm formation. These proteins included nucleoside diphosphate sugar epimerases (WG8-12747/10461) that are involved in the glycosylation of the cell surface and were previously detected in a proteomic study of Halorubrum lacusprofundi biofilm formation (32). Both tagaturonate epimerase (WG8-10872) and glucuronate isomerase (WG8-10873) are involved in converting hexuronic acids to fructuronate and subsequently fructuronate to glucuronate, which is a known substrate in biofilm exopolymer synthesis in Lactobacillus casei, Streptococcus thermophilus, and Pseudomonas aeruginosa (33). Translocation and assembly module TamB (WG8-10736) is a membrane protein involved in the secretion of adhesion proteins that promote biofilm formation in E. coli (34). Multiple outer membrane TonB-associated proteins (WG8-102136, -10246, -10245, -10244, -10243, -102135, -102134, and -101121) involved in large-molecule movement across the membrane were also present at higher abundances in high-pressure-grown cells. These proteins can transport molecules including carbohydrates, metals, and quorum-sensing signaling molecules (30, 31, 57) and have been implicated in stimulating biofilm formation in P. aeruginosa, Thermotoga maritima, and Staphylococcus aureus (29–31). Glucose-1-phosphate adenylyltransferase (WG8-10557/10556) and 1,4-alpha-glucan branching enzyme (WG8-10558) were both present in greater abundances under pressurized growth conditions and are involved in cellular glycogen synthesis. Studies with known biofilm-forming microorganisms (e.g., Salmonella enterica serovar Enteritidis) have demonstrated that these microorganisms accumulate intracellular glycogen to help in EPS production (26). Supporting our inference that *Halanaerobium* may utilize a similar mechanism for EPS generation, a pullulanase enzyme (WG8-10553) needed to hydrolyze starch linkages for EPS biosynthesis was more abundant at high pressure (36) (see Fig. S1 in the supplemental material).

Other proteins associated with WG8 growth at high pressure that may play roles in EPS formation include mannonate dehydratase (WG8-11618/10874), which was upregulated in Enterococcus faecium biofilms (20), and maltose phosphorylase (WG8-103108), which is involved in the conversion of maltose to glucose. This maltose-to-glucose conversion is of interest because glucose has been found to enhance biofilm formation in Staphylococcus epidermidis (27). Other high-pressure-associated proteins include phosphofructokinase (WG8-11516/10552), which has been detected at higher abundances in Streptococcus mutans biofilms (24), and phosphomannomutase (WG8-11441/11253), which is associated with exopolysaccharide biosynthesis in Pseudomonas aeruginosa (23). The l-fucose isomerase enzyme (WG8-10560) plays a key role in production of l-fucose, a known component of a tetrasaccharide repeat in Klebsiella pneumoniae and Enterobacter aerogenes biofilms (58). *N*-Acetylglucosamine-6-phosphate deacetylase (WG8-103102) is involved in the synthesis of the alginate precursor fructose-6-phosphate, an adhesive component of P. aeruginosa biofilms (59).

Finally, proteins associated with the phosphoenolpyruvate phosphotransferase system and other sugar transport systems (WG8-11518, -11087, -102110, and -102109) have been shown to play regulatory roles in biofilm formation in Vibrio cholerae and Thermotoga maritima (22, 29). Other regulatory proteins include the sigma-54 modulation protein (WG8-1072), which has been found to control biofilm development of Vibrio fischeri (25), and host factor I protein (WG8-102151), which plays a role in regulation of sigma factor RpoS, a master regulator of biofilm formation that is utilized during high-pressure growth in E. coli (28, 47).

All the proteins highlighted above provide strong evidence that H. congolense WG8 is capable of forming biofilm-like structures under pressures representative of the deep subsurface. Complementing these inferences, CLSM analysis of biomass revealed greater cell aggregation and production of EPS-like material in high-pressure incubations. We hypothesize the increased EPS formation is a *Halanaerobium* WG8 stress response, as indicated by lower growth rates at increased pressures. Other studies have grown E. coli in pressurized microfluidic devices and found that the mechanical stress associated with living in tightly packed environment induced a biochemical stress response that included EPS generation and biofilm formation (60). Biofilm and EPS-associated structures could potentially impact hydrocarbon recovery from fractured shales; fractures within the shale matrix are nanometers to centimeters in size (1, 61), and bioclogging associated with EPS production by *Halanaerobium* strains could potentially reduce the permeability of the system. Increased EPS production and formation of biofilm-like structures could also impact the efficacy of biocides that are injected into the target formation. Indeed, prior research has suggested that EPS-type materials can offer protection to microorganisms against a wide range of environmental stresses, including host immune defenses (62), UV radiation, supercritical carbon dioxide (9), and biocides (63, 64). It is possible that the stress response induced by high pressure may have the unintended advantage of offering *Halanaerobium* increased protection from added biocides. EPS-related biocide resistance may at least partly explain the observed persistence of microbial consortia, which include *Halanaerobium*, within hydraulically fractured shales for multiple years following the fracturing process (2, 3, 65–67).

### Conclusions.

H. congolense WG8 is a piezotolerant microorganism that is characteristic of *Halanaerobium* strains that dominate microbial ecosystems in hydraulically fractured shales. The metabolic and physiological responses to the onset of high-pressure growth conditions include the (i) inferred production of increased EPS that drives cell aggregation and (ii) rearrangement of central metabolism such that production of organic acids and alcohols is favored over that of hydrogen. Both of these responses could drive potentially deleterious processes in the subsurface, such as bioclogging of newly generated fracture networks and pores and increased rates of corrosion of carbon steel infrastructure associated with hydrocarbon recovery. Additionally, the increased activity of diol dehydratases under high-pressure conditions highlights the metabolic plasticity and versatility of *Halanaerobium* under rapidly changing environmental conditions and may contribute to *in situ* degradation of chemical additives used in the hydraulic fracturing process. Together, these results highlight the importance of studying microbial physiology and metabolism under representative environmental conditions and stress the importance of microbial control in hydraulically fractured shales.

## MATERIALS AND METHODS

### *Halanaerobium* growth experiments.

Halanaerobium congolense WG8 was isolated from a produced water sample from the Utica shale, as described previously (5), and draft genome sequenced at the Joint Genome Institute using Illumina HiSeq technology. Growth curves were performed in triplicate at 0.1, 21, 35, and 48 MPa, with optical density (OD) measurements collected every 24 h. Biomass was incubated at 40°C in anaerobic Hungate tubes (99% N_2_ headspace) containing 9 ml of saltwater liquid medium (described by Booker et al. [5]) inoculated with 10% *Halanaerobium* WG8 growing in mid-log phase (5). Tubes were modified per the protocol outlined by Bowles et al. (68) so that they could be pressurized within titanium pressure vessels manufactured by the Marine Science Development Shop at Scripps Institution of Oceanography. Water was used as a pressurizing phase in these reactors. To generate standard growth curves that related *Halanaerobium* optical density to cells per milliliter, optical density (600 nm) measurements and 500 µl of culture were collected every 24 h from each culture tube until stationary growth phase was reached. Each 500 µl of culture collected was vortexed to disperse cells for more accurate optical density measurements. Two 10-µl samples were taken from the 500-µl aliquot and were counted using a hemocytometer. These counts were used to calculate the number of cells present in the growth culture and correlate them to optical density (see Fig. S2 in the supplemental material). Growth rates were calculated using ln OD taken during the period of exponential growth, while cell yield was inferred from the highest optical density reading and corresponding cell counts (69).

### ^1^H-NMR measurements of fermentation products.

Biological triplicate cell cultures pressurized at 0.1 and 35 MPa were collected during mid-log growth phase. Supernatant was filtered through a 0.22-µm filter, flash frozen, and shipped to the Environmental Molecular Sciences Laboratory (EMSL) for metabolite quantification using proton NMR (^1^H-NMR). The one-dimensional (1D) ^1^H-NMR spectra of all samples were collected following standard Chenomx (Edmonton, AB, Canada) sample preparation and data collection guidelines (70). Biological triplicate data were acquired on a Varian Direct Drive (VNMRS) 600-MHz spectrometer (Agilent Technologies) equipped with a Varian triple-resonance salt-tolerant cold probe with a cold carbon preamplifier. A Varian standard one-dimensional proton nuclear Overhauser effect spectrum (NOESY) with presaturation (TNNOESY) was collected on each sample, using the Chenomx standard data collection protocol (70). Collected spectra were analyzed using Chenomx 8.3 software, with quantifications based on spectral intensities relative to a calibrated reference solution (100% D_2_O, 0.5 mM 2,2-dimethyl-2-silapentane-5-sulfonate-*d*6 [DSS]), as previously described (2).

### GC.

For gas chromatography (GC), biological triplicates of *Halanaerobium* WG8 were grown at 0.1 and 35 MPa until mid-log phase was reached. Samples for gas production were taken at the beginning of lag phase and once mid-log phase was reached. Cultures grown at 35 MPa were transferred into 20-ml vacuum-sealed bottles. All samples were shaken for 1 h at 170 rpm to allow soluble H_2_ and CO_2_ to become gaseous. We acknowledge that this method does not guarantee that all soluble H_2_ and CO_2_ becomes gaseous, and therefore our CO_2_ and H_2_ measurements likely underestimate the production of these gases by Halanaerobium congolense WG8. After shaking, each sample was inverted and stored at 4°C overnight. To measure the concentration of carbon dioxide and hydrogen gas associated with each sample, 5 ml of headspace was sampled and analyzed using a GC-2014 Shimadzu gas chromatograph. The measured peak area was converted to moles using the density and molecular weight of each gas.

### Proteomics sample preparation.

Total protein profiles of *Halanaerobium* grown at 0.1 and 35 MPa were determined using shotgun proteomics. Triplicate biomass from growth experiments at 0.1 and 35 MPa was harvested at mid-log phase by centrifugation at 10,000 rpm and 4°C for 10 min. The cell pellets were immediately flash frozen in liquid nitrogen to preserve protein signatures. Sample preparation for proteomic analysis was previously described by Booker et al. (5). Briefly, total proteins were extracted from each cell pellet using an extraction kit (Expedeon, San Diego, CA) and digested in 0.05 μM trypsin. The resulting peptides were filtered, concentrated, and diluted to 0.3 µg/µL for MS analysis.

### Proteomics measurements.

MS analysis of peptide mixtures was previously described by Booker et al. (5). Briefly, peptide mixtures were separated using a 2D-LC Acquity ultraperformance liquid chromatography (UPLC) M-Class system (Waters, Milford, MA) with a silica hand-packed column with 3-µm particle Jupiter C_18_ derivatized silica beads (Phenomenex, Torrance, CA). Mobile phases consisted of 0% to 100% acetonitrile–0.1% formic acid against water–0.1% formic acid. This LC system was coupled to an in-house-built nanoelectrospray apparatus. MS analyses were performed using a Thermo Fisher (Framingham, MA) QExactive Pro, and measured peptides were searched against predicted peptides derived from the H. congolense WG8 genome. The resulting peptide identifications were filtered via MSGF Q-Value ≤ 0.01, which is an ∼1% false-discovery rate (FDR) at each individual data set level. There were 7,672 reversed identifications out of 813,177 total filter passing identifications, for a 0.94% FDR at the peptide-to-spectrum match (PSM) level. For comparative analyses between triplicate biological replicates, protein spectral counts were normalized using the normalized spectral abundance frequency (NSAF) method (71), and Z-score values were calculated to display differences in protein abundances. Significant differences in protein abundances across incubation conditions were determined using a two-tailed Student *t* test with unpaired equal variance across triplicate NSAF values, with resulting *P* values of 0.05 or below indicating significance.

### PLFA analysis.

Culture samples were extracted ultrasonically according to the modified Bligh-Dyer procedure (72, 73) after adding an intact polar lipid (phosphate buffer plus phosphatidylcholine [POPC]). Total lipid extracts (TLEs) were transferred into test tubes using three washes of 2 ml of chloroform, after which the solvent was evaporated with N_2_ at 37°C. Dried TLEs were resuspended in 2 ml of chloroform and fractionated using silicic acid chromatography, with phospholipid fatty acids (PLFAs) recovered from methanol. Extracts were next evaporated to dryness before methylation using methanolic potassium hydroxide (73, 74). Fatty acid methyl esters (FAMEs) were next dissolved in 200 μl of hexane containing 50 pmol/μl of external injection standard (docosanoic acid methyl ester; Matreya, Inc.) and transferred into GC-MS vials containing 500-µl glass inserts. Sample aliquots were injected into an Agilent 6890 series gas chromatograph (GC) interfaced to an Agilent 5973 mass selective detector (MS) equipped with a nonpolar cross-linked methyl silicone column (Restek RTX-1 column; 60 m, 0.25-mm inner diameter, 0.25-µm film thickness). GC operating conditions were as follows: 60°C for 2 min, then increased at 10°C/min to 150°C, followed by a second ramp at 3°C/min to 312°C, for a total run time of 65 min (75). The injector temperature was 230°C, the detector temperature was 300°C, and helium was the carrier gas. The following methyl ester standards (Matreya LLC, State College, PA, USA) were included in each sample run to calibrate retention times and assist with peak identification: bacterial acid methyl ester CP mixture (BacFAME [1114]), polyunsaturated FAME mixture 2 (PUFA-2 [1081]), and polyunsaturated FAME mixture 3 (PUFA-3 [1177]). Identified peaks were confirmed across all samples, with GC-MS spectra validated using Agilent MSD ChemStation data analysis software F.01.00 with the NIST11 compound library. A single-ion monitoring program was also used to scan the base peaks for lipids to validate all identified peaks. Once peaks were identified, the lipid concentration was calculated based on external standard peak area. An internal standard curve ranging from 1 to 50 pmol/µl was used to determine the detection limit and establish the sample dilution range. Lipid extraction and GC-MS analysis were performed at the Center for Environmental Biotechnology at the University of Tennessee (Pfiffner Lab, Knoxville, TN, USA).

### CLSM.

*Halanaerobium* cultures were incubated at 0.1 and 35 MPa in biological quadruplicates for 72 h at 40°C. After incubation, cells were prepared for confocal laser scanning microscopy (CLSM) imaging at the Ohio State University Molecular and Cellular Imaging Center in Wooster, OH. The bottom 2 ml of the cell cultures were fixed by adding an equal volume of 8% paraformaldehyde in 200 mM Tris-HCl (pH 7.2) buffer and incubated at 4°C overnight without shaking. Cells were collected by centrifugation at 1,000 × *g* for 10 min, resuspended in 200 μl of 10 mM Tris-HCl (pH 7.2), and then stained. Cells were stained with 50 μg/ml of concanavalin A (ConA)-Alexa Fluor 488 (Invitrogen, catalog no. C11252) for 40 min to visualize α-mannopyranosyl and α-glucopyranosyl residues (green) within the extracellular polymeric substance matrix and with 1 μM of Syto59 (Invitrogen, catalog no. 11341) for 30 min to visualize nucleic acids (red). After staining, cells were collected by centrifugation, washed once with 200 μl of 10 mM Tris-HCl (pH 7.2), and resuspended in 15 μl of 10 mM Tris-HCl (pH 7.2). Samples were immediately mounted on a glass slide and imaged on a Leica TCS-SP6 confocal microscope. For the quantification of the green fluorescence (extracellular polymeric matrix), stacks (average projections) of seven focal planes (z = 24 μm) were acquired using a 63×/1.20 water objective. A total of 20 images (five images from four separate slides) for each growing condition were collected. Gray pixel values for each image were acquired using ImageJ, and the total green fluorescence was calculated from the integrated density for each image, adjusted for the background fluorescence values. Values from each sample were averaged, and total green fluorescence (EPS) was plotted for *Halanaerobium* grown at both 0.1 and 35 MPa.

### Data availability.

The genome of Halanaerobium congolense WG8 was sequenced and annotated by the Joint Genome Institute and is publicly available in the JGI Genome Portal database (http://genome.jgi.doe.gov/) under IMG number 2642422587.

## Supplementary Material

Supplemental file 1

Supplemental file 2

Supplemental file 3

Supplemental file 4

## References

[1] Zhang P, Hu L, Meegoda JN, Gao S 2015 Micro/nano-pore network analysis of gas flow in shale matrix. Sci Rep 5:13501. doi:10.1038/srep13501. 26310236 PMC4642512

[2] Daly RA, Borton MA, Wilkins MJ, Hoyt DW, Kountz DJ, Wolfe RA, Welch SA, Marcus DN, Trexler RV, MacRae JD, Krzycki JA, Cole DR, Mouser PJ, Wrighton KC 2016 Microbial metabolisms in a 2.5-km-deep ecosystem created by hydraulic fracturing in shales. Nat Microbiol 1:16146. doi:10.1038/nmicrobiol.2016.146. 27595198

[3] Cluff MA, Hartsock A, MacRae JD, Carter K, Mouser PJ 2014 Temporal changes in microbial ecology and geochemistry in produced water from hydraulically fractured marcellus shale gas wells. Environ Sci Technol 48:6508–6517. doi:10.1021/es501173p. 24803059

[4] Mouser PJ, Borton M, Darrah TH, Hartsock A, Wrighton KC 2016 Hydraulic fracturing offers view of microbial life in the deep terrestrial subsurface. FEMS Microbiol Ecol 92:fiw166. doi:10.1093/femsec/fiw166. 27507739

[5] Booker AE, Borton MA, Daly RA, Welch SA, Nicora CD, Hoyt DW, Wilson T, Purvine SO, Wolfe RA, Sharma S, Mouser PJ, Cole DR, Lipton MS, Wrighton KC, Wilkins MJ 2017 Sulfide generation by dominant *Halanaerobium* microorganisms in hydraulically fractured shales. mSphere 2:e00257-17. doi:10.1128/mSphereDirect.00257-17. 28685163 PMC5497025

[6] Lipus D, Vikram A, Ross D, Bain D, Gulliver D, Hammack R, Bibby K 2017 Predominance and metabolic potential of *Halanaerobium* spp. in produced water from hydraulically fractured marcellus shale wells. Appl Environ Microbiol 83:e02659-16. doi:10.1128/AEM.02659-16. 28159795 PMC5377500

[7] Liang R, Davidova IA, Marks CR, Stamps BW, Harriman BH, Stevenson BS, Duncan KE, Suflita JM 2016 Metabolic capability of a predominant *Halanaerobium* sp. in hydraulically fractured gas wells and its implication in pipeline corrosion. Front Microbiol 7:988. doi:10.3389/fmicb.2016.00988. 27446028 PMC4916785

[8] Borton MA, Hoyt DW, Roux S, Daly RA, Welch SA, Nicora CD, Purvine SO, Eder EK, Hanson AJ, Sheets JM, Morgan DM, Sharma S, Carr TR, Cole DR, Mouser PJ, Lipton MS, Wilkins MJ, Wrighton KC 2018 Coupled laboratory and field investigations resolve microbial interactions that underpin persistence in hydraulically fractured shales. Proc Natl Acad Sci USA 115:E6585–E6594. doi:10.1073/pnas.1800155115. 29941576 PMC6048472

[9] Mitchell AC, Phillips AJ, Hamilton MA, Gerlach R, Hollis WK, Kaszuba JP, Cunningham AB 2008 Resilience of planktonic and biofilm cultures to supercritical CO2. J Supercrit Fluids 47:318–325. doi:10.1016/j.supflu.2008.07.005.

[10] Rosenberg E, DeLong EF, Lory S, Stackebrandt E, Thompson F 2013 The prokaryotes: prokaryotic communities and ecophysiology. Springer, Berlin, Germany.

[11] Picard A, Daniel I 2013 Pressure as an environmental parameter for microbial life—a review. Biophys Chem 183:30–41. doi:10.1016/j.bpc.2013.06.019. 23891571

[12] Kish A, Griffin PL, Rogers KL, Fogel ML, Hemley RJ, Steele A 2012 High-pressure tolerance in Halobacterium salinarum NRC-1 and other non-piezophilic prokaryotes. Extremophiles 16:355–361. doi:10.1007/s00792-011-0418-8. 22212652

[13] Ragsdale SW 2003 Pyruvate ferredoxin oxidoreductase and its radical intermediate. Chem Rev 103:2333–2346. doi:10.1021/cr020423e. 12797832

[14] Zhang Y, Li X, Bartlett DH, Xiao X 2015 Current developments in marine microbiology: high-pressure biotechnology and the genetic engineering of piezophiles. Curr Opin Biotechnol 33:157–164. doi:10.1016/j.copbio.2015.02.013. 25776196

[15] Welch TJ, Farewell A, Neidhardt FC, Bartlett DH 1993 Stress response of Escherichia coli to elevated hydrostatic pressure. J Bacteriol 175:7170–7177. doi:10.1128/jb.175.22.7170-7177.1993. 8226663 PMC206858

[16] Resch A, Rosenstein R, Nerz C, Gotz F 2005 Differential gene expression profiling of Staphylococcus aureus cultivated under biofilm and planktonic conditions. Appl Environ Microbiol 71:2663–2676. doi:10.1128/AEM.71.5.2663-2676.2005. 15870358 PMC1087559

[17] Sinha S, Cheng S, Fan C, Bobik TA 2012 The PduM protein is a structural component of the microcompartments involved in coenzyme B12-dependent 1,2-propanediol degradation by Salmonella enterica. J Bacteriol 194:1912–1918. doi:10.1128/JB.06529-11. 22343294 PMC3318458

[18] Roux D, Cywes-Bentley C, Zhang Y-F, Pons S, Konkol M, Kearns DB, Little DJ, Howell PL, Skurnik D, Pier GB 2015 Identification of Poly-N-acetylglucosamine as a major polysaccharide component of the Bacillus subtilis biofilm matrix. J Biol Chem 290:19261–19272. doi:10.1074/jbc.M115.648709. 26078454 PMC4521046

[19] Hobley L, Li B, Wood JL, Kim SH, Naidoo J, Ferreira AS, Khomutov M, Khomutov A, Stanley-Wall NR, Michael AJ 2017 Spermidine promotes Bacillus subtilis biofilm formation by activating expression of the matrix regulator slrR. J Biol Chem 292:12041–12053. doi:10.1074/jbc.M117.789644. 28546427 PMC5519356

[20] Lim SY, Teh CSJ, Thong KL 2017 Biofilm-related diseases and omics: global transcriptional profiling of Enterococcus faecium reveals different gene expression patterns in the biofilm and planktonic cells. Omics 21:592–602. doi:10.1089/omi.2017.0119. 29049010

[21] Gil C, Solano C, Burgui S, Latasa C, García B, Toledo-Arana A, Lasa I, Valle J 2014 Biofilm matrix exoproteins induce a protective immune response against Staphylococcus aureus biofilm infection. Infect Immun 82:1017–1029. doi:10.1128/IAI.01419-13. 24343648 PMC3957983

[22] Houot L, Chang S, Pickering BS, Absalon C, Watnick PI 2010 The phosphoenolpyruvate phosphotransferase system regulates Vibrio cholerae biofilm formation through multiple independent pathways. J Bacteriol 192:3055–3067. doi:10.1128/JB.00213-10. 20400550 PMC2901703

[23] Wei Q, Ma L 2013 Biofilm matrix and its regulation in Pseudomonas aeruginosa. Int J Mol Sci 14:20983–21005. doi:10.3390/ijms141020983. 24145749 PMC3821654

[24] Welin J, Wilkins JC, Beighton D, Svensater G 2004 Protein expression by Streptococcus mutans during initial stage of biofilm formation. Appl Environ Microbiol 70:3736–3741. doi:10.1128/AEM.70.6.3736-3741.2004. 15184181 PMC427790

[25] Wolfe AJ, Millikan DS, Campbell JM, Visick KL 2004 Vibrio fischeri 54 controls motility, biofilm formation, luminescence, and colonization. Appl Environ Microbiol 70:2520–2524. doi:10.1128/AEM.70.4.2520-2524.2004. 15066853 PMC383144

[26] Bonafonte MA, Solano C, Sesma B, Alvarez M, Montuenga L, García-Ros D, Gamazo C 2000 The relationship between glycogen synthesis, biofilm formation and virulence in Salmonella enteritidis. FEMS Microbiol Lett 191:31–36. doi:10.1111/j.1574-6968.2000.tb09315.x. 11004396

[27] Mack D, Siemssen N, Laufs R 1992 Parallel induction by glucose of adherence and a polysaccharide antigen specific for plastic-adherent Staphylococcus epidermidis: evidence for functional relation to intercellular adhesion. Infect Immun 60:2048–2057. 1314224 10.1128/iai.60.5.2048-2057.1992 PMC257114

[28] Becker A 2016 Classic spotlight: Hfq, from a specific host factor for phage replication to a global player in riboregulation. J Bacteriol 198:2279–2280. doi:10.1128/JB.00472-16. 27514488 PMC4984557

[29] Pysz MA, Conners SB, Clemente I, Shockley KR, Johnson MR, Donald E, Kelly RM, Montero CI, Ward DE 2004 Transcriptional analysis of biofilm formation processes in the anaerobic, hyperthermophilic bacterium Thermotoga maritima. Appl Environ Microbiol 70:6098–6112. doi:10.1128/AEM.70.10.6098-6112.2004. 15466556 PMC522082

[30] Abbas A, Adams C, Scully N, Glennon J, O'Gara F 2007 A role for TonB1 in biofilm formation and quorum sensing in Pseudomonas aeruginosa. FEMS Microbiol Lett 274:269–278. doi:10.1111/j.1574-6968.2007.00845.x. 17623027

[31] Lin MH, Shu JC, Huang HY, Cheng YC 2012 Involvement of iron in biofilm formation by staphylococcus aureus. PLoS One 7:e34388. doi:10.1371/journal.pone.0034388. 22479621 PMC3313993

[32] Liao Y, Williams TJ, Ye J, Charlesworth J, Burns BP, Poljak A, Raftery MJ, Cavicchioli R 2016 Morphological and proteomic analysis of biofilms from the Antarctic archaeon, Halorubrum lacusprofundi. Sci Rep 6:37454. doi:10.1038/srep37454. 27874045 PMC5118699

[33] Sauer K, Camper AK, Ehrlich GD, Costerton JW, Davies DG 2002 Pseudomonas aeruginosa displays multiple phenotypes during development as a biofilm. J Bacteriol 184:1140–1154. doi:10.1128/jb.184.4.1140-1154.2002. 11807075 PMC134825

[34] Selkrig J, Mosbahi K, Webb CT, Belousoff MJ, Perry AJ, Wells TJ, Morris F, Leyton DL, Totsika M, Phan M-D, Celik N, Kelly M, Oates C, Hartland EL, Robins-Browne RM, Ramarathinam SH, Purcell AW, Schembri MA, Strugnell RA, Henderson IR, Walker D, Lithgow T 2012 Discovery of an archetypal protein transport system in bacterial outer membranes. Nat Struct Mol Biol 19:506–510. doi:10.1038/nsmb.2261. 22466966

[35] Li Y, Cao S, Zhang L, Yuan J, Lau GW, Wen Y, Wu R, Zhao Q, Huang X, Yan Q, Huang Y, Wen X 2016 Absence of TolC impairs biofilm formation in Actinobacillus pleuropneumoniae by reducing initial attachment. PLoS One 11:e0163364. doi:10.1371/journal.pone.0163364. 27681876 PMC5040401

[36] Hii SL, Tan JS, Ling TC, Ariff AB 2012 Pullulanase: role in starch hydrolysis and potential industrial applications. Enzyme Res 2012:1–14. doi:10.1155/2012/921362. PMC3443597 22991654

[37] Whiteley CG, Lee D-J 2015 Bacterial diguanylate cyclases: structure, function and mechanism in exopolysaccharide biofilm development. Biotechnol Adv 33:124–141. doi:10.1016/j.biotechadv.2014.11.010. 25499693

[38] de Sarrau B, Clavel T, Clerté C, Carlin F, Giniès C, Nguyen-The C 2012 Influence of anaerobiosis and low temperature on Bacillus cereus growth, metabolism, and membrane properties. Appl Environ Microbiol 78:1715–1723. doi:10.1128/AEM.06410-11. 22247126 PMC3298147

[39] Scoma A, Heyer R, Rifai R, Dandyk C, Marshall I, Kerckhof F-M, Marietou A, Boshker HTS, Meysman FJR, Malmos KG, Vosegaard T, Vermeir P, Banat IM, Benndorf D, Boon N 2019 Reduced TCA cycle rates at high hydrostatic pressure hinder hydrocarbon degradation and obligate oil degraders in natural, deep-sea microbial communities. ISME J 13:1004–1018. doi:10.1038/s41396-018-0324-5. 30542078 PMC6461773

[40] Mingeot-Leclercq M-P, Décout J-L 2016 Bacterial lipid membranes as promising targets to fight antimicrobial resistance, molecular foundations and illustration through the renewal of aminoglycoside antibiotics and emergence of amphiphilic aminoglycosides. Med Chem Commun (Camb) 7:586–611. doi:10.1039/C5MD00503E.

[41] Allen EE, Facciotti D, Bartlett DH 1999 Monounsaturated but not polyunsaturated fatty acids are required for growth of the deep-sea bacterium Photobacterium profundum SS9 at high pressure and low temperature. Appl Environ Microbiol 65:1710–1720. 10103272 10.1128/aem.65.4.1710-1720.1999 PMC91242

[42] Wang F, Xiao X, Ou H-Y, Gai Y, Wang F 2009 Role and regulation of fatty acid biosynthesis in the response of Shewanella piezotolerans WP3 to different temperatures and pressures. J Bacteriol 191:2574–2584. doi:10.1128/JB.00498-08. 19201790 PMC2668426

[43] Sollich M, Yoshinaga MY, Häusler S, Price RE, Hinrichs K-U, Bühring SI 2017 Heat stress dictates microbial lipid composition along a thermal gradient in marine sediments. Front Microbiol 8:1550. doi:10.3389/fmicb.2017.01550. 28878741 PMC5572230

[44] Fang J, Kato C 2010 Deep-sea piezophilic bacteria: geomicrobiology and biotechnology. *In* Jain S, Khan A, Rai M (ed), Geomicrobiology, 1st ed CRC Press, Boca Raton, FL.

[45] Biswas R, Zheng T, Olson DG, Lynd LR, Guss AM 2015 Elimination of hydrogenase active site assembly blocks H2 production and increases ethanol yield in Clostridium thermocellum. Biotechnol Biofuels 8:20. doi:10.1186/s13068-015-0204-4. 25763101 PMC4355364

[46] Michoud G, Jebbar M 2016 High hydrostatic pressure adaptive strategies in an obligate piezophile Pyrococcus yayanosii. Sci Rep 6:27289. doi:10.1038/srep27289. 27250364 PMC4890121

[47] Bartlett DH 2002 Pressure effects on in vivo microbial processes. Biochim Biophys Acta 1595:367–381. doi:10.1016/S0167-4838(01)00357-0. 11983409

[48] Pletcher D, Sidorin D, Hedges B 2007 Acetate-enhanced corrosion of carbon steel—further factors in oilfield environments. Corrosion 63:285–294. doi:10.5006/1.3278356.

[49] Schmid G, Lüdemann HD, Jaenicke R 1975 High pressure effects on the activity of glycolytic enzymes. Biophys Chem 3:90–98. doi:10.1016/0301-4622(75)80041-X. 1092383

[50] Bobik TA 2006 Polyhedral organelles compartmenting bacterial metabolic processes. Appl Microbiol Biotechnol 70:517–525. doi:10.1007/s00253-005-0295-0. 16525780

[51] Daniel R, Bobik T, Gottschalk G 1998 Biochemistry of coenzyme B12-dependent glycerol and diol dehydratases and organization of the encoding genes. FEMS Microbiol Rev 22:553–566. doi:10.1111/j.1574-6976.1998.tb00387.x. 9990728

[52] Smith DM, Golding BT, Radom L 2001 Understanding the mechanism of B12-dependent diol dehydratase: a synergistic retro-push−pull proposal. J Am Chem Soc 123:1664–1675. doi:10.1021/ja001454z. 11456766

[53] Collas F, Kuit W, Clément B, Marchal R, López-Contreras AM, Monot F 2012 Simultaneous production of isopropanol, butanol, ethanol and 2,3-butanediol by Clostridium acetobutylicum ATCC 824 engineered strains. AMB Express 2:45. doi:10.1186/2191-0855-2-45. 22909015 PMC3583297

[54] Trifunović D, Schuchmann K, Müller V 2016 Ethylene glycol metabolism in the acetogen *Acetobacterium woodii*. J Bacteriol 198:1058–1065. doi:10.1128/JB.00942-15. 26787767 PMC4800866

[55] Schuchmann K, Müller V 2014 Autotrophy at the thermodynamic limit of life: a model for energy conservation in acetogenic bacteria. Nat Rev Microbiol 12:809–821. doi:10.1038/nrmicro3365. 25383604

[56] Xie Z, Jian H, Jin Z, Xiao X 2018 Enhancing the adaptability of the deep-sea bacterium Shewanella piezotolerans WP3 to high pressure and low temperature by experimental evolution under H_2_O_2_ stress. Appl Environ Microbiol 84:e02342-17. doi:10.1128/AEM.02342-17. 29269502 PMC5812942

[57] Noinaj N, Guillier M, Barnard TJ, Buchanan SK 2010 TonB-dependent transporters: regulation, structure, and function. Annu Rev Microbiol 64:43–60. doi:10.1146/annurev.micro.112408.134247. 20420522 PMC3108441

[58] Morikawa M, Kagihiro S, Haruki M, Takano K, Branda S, Kolter R, Kanaya S 2006 Biofilm formation by a Bacillus subtilis strain that produces gamma-polyglutamate. Microbiology 152:2801–2807. doi:10.1099/mic.0.29060-0. 16946274

[59] Rehm BHA 2009 Alginates: biology and applications. Springer, Berlin, Germany.

[60] Chu EK, Kilic O, Cho H, Groisman A, Levchenko A 2018 Self-induced mechanical stress can trigger biofilm formation in uropathogenic Escherichia coli. Nat Commun 9:4087. doi:10.1038/s41467-018-06552-z. 30291231 PMC6173693

[61] Gu X, Cole DR, Rother G, Mildner DFR, Brantley SL 2015 Pores in marcellus shale: a neutron scattering and FIB-SEM study. Energy Fuels 29:1295–1308. doi:10.1021/acs.energyfuels.5b00033.

[62] Wozniak DJ, Limoli DH, Jones CJ 2015 Bacterial extracellular polysaccharides in biofilm formation and function, p 223–247. *In* Ghannoum M, Parsek M, Whiteley M, Mukherjee P (ed), Microbial biofilms, 2nd ed American Society for Microbiology, Washington, DC. 10.1128/microbiolspec.MB-0011-2014 PMC4657554 26185074

[63] Mah TF, O'Toole GA 2001 Mechanisms of biofilm resistance to antimicrobial agents. Trends Microbiol 9:34–39. doi:10.1016/S0966-842X(00)01913-2. 11166241

[64] Epstein AK, Pokroy B, Seminara A, Aizenberg J 2011 Bacterial biofilm shows persistent resistance to liquid wetting and gas penetration. Proc Natl Acad Sci U S A 108:995–1000. doi:10.1073/pnas.1011033108. 21191101 PMC3024672

[65] Davis JP, Struchtemeyer CG, Elshahed MS 2012 Bacterial communities associated with production facilities of two newly drilled thermogenic natural gas wells in the Barnett Shale (Texas, USA). Microb Ecol 64:942–954. doi:10.1007/s00248-012-0073-3. 22622766

[66] Murali Mohan A, Hartsock A, Bibby KJ, Hammack RW, Vidic RD, Gregory KB 2013 Microbial community changes in hydraulic fracturing fluids and produced water from shale gas extraction. Environ Sci Technol 47:13141–13150. doi:10.1021/es402928b. 24088205

[67] Wuchter C, Banning E, Mincer TJ, Drenzek NJ, Coolen M 2013 Microbial diversity and methanogenic activity of Antrim Shale formation waters from recently fractured wells. Front Microbiol 4:367. doi:10.3389/fmicb.2013.00367. 24367357 PMC3853793

[68] Bowles MW, Samarkin VA, Joye SB 2011 Improved measurement of microbial activity in deep-sea sediments at in situ pressure and methane concentration. Limnol Oceanogr Methods 9:499–506. doi:10.4319/lom.2011.9.499.

[69] Hall BG, Acar H, Nandipati A, Barlow M 2014 Growth rates made easy. Mol Biol Evol 31:232–238. doi:10.1093/molbev/mst187. 24170494

[70] Weljie AM, Newton J, Mercier P, Carlson E, Slupsky CM 2006 Targeted profiling: quantitative analysis of 1 H NMR metabolomics data. Anal Chem 78:4430–4442. doi:10.1021/ac060209g. 16808451

[71] Paoletti AC, Parmely TJ, Tomomori-Sato C, Sato S, Zhu D, Conaway RC, Conaway JW, Florens L, Washburn MP 2006 Quantitative proteomic analysis of distinct mammalian Mediator complexes using normalized spectral abundance factors. Proc Natl Acad Sci U S A 103:18928–18933. doi:10.1073/pnas.0606379103. 17138671 PMC1672612

[72] Bligh EG, Dyer WJ 1959 A rapid method of total lipid extraction and purification. Can J Biochem Physiol 37:911–917. doi:10.1139/o59-099. 13671378

[73] Ringelberg DB, Sutton S, White DC 1997 Biomass, bioactivity and biodiversity: microbial ecology of the deep subusrface: analysis of ester-linked phospholipid fatty acids. FEMS Microbiol Rev 20:371–377. doi:10.1111/j.1574-6976.1997.tb00322.x.

[74] Kieft TL, Ringelberg DB, White DC 1994 Changes in ester-linked phospholipid fatty acid profiles of subsurface bacteria during starvation and desiccation in a porous medium. Appl Environ Microbiol 60:3292–3299. 16349382 10.1128/aem.60.9.3292-3299.1994 PMC201801

[75] White DC, Ringelberg DB 1997 Signature lipid biomarker analysis, p 225–272. *In* Burlage RS, Atlas R, Stahl D, Geesey G, Sayler G (ed), Techniques in microbial ecology. Oxford University Press, New York, NY.

