# Supplementary material for: Deep-Subsurface Pressure Stimulates Metabolic Plasticity in Shale-Colonizing Halanaerobium spp
Source: Appl Environ Microbiol. 2019 May 30;85(12):e00018-19. doi: 10.1128/AEM.00018-19 (PMC6544827; doi:10.1128/AEM.00018-19)
Supplement: Supplemental file 1 [file AEM.00018-19-s0001.pdf]

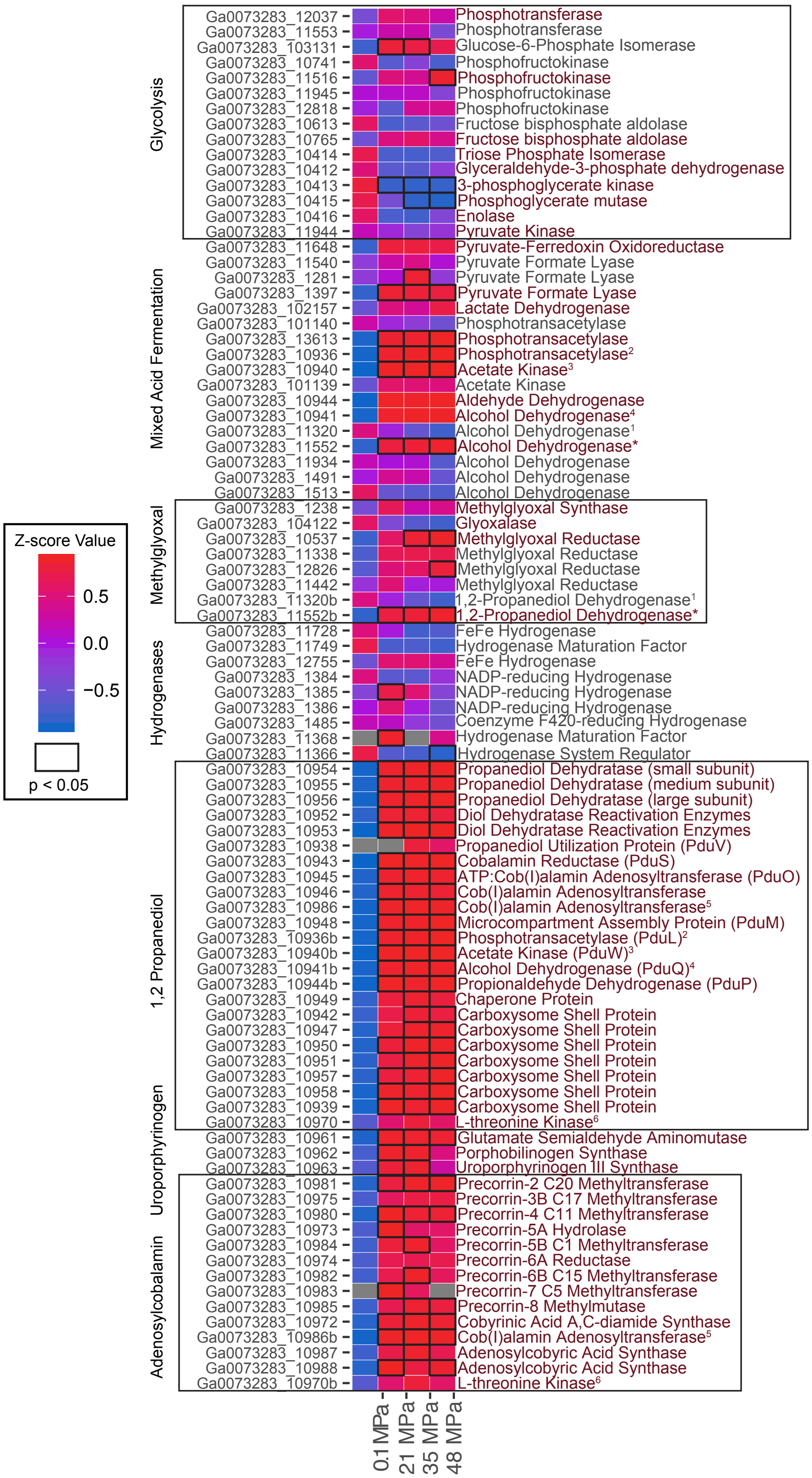

Z-score Value
0.5
0.0
−0.5
p < 0.05
Glycolysis
Mixed Acid Fermentation
Methylglyoxal
Hydrogenases
1,2 Propanediol
Uroporphyrinogen
Adenosylcobalamin
Ga0073283_12037 Phosphotransferase
Ga0073283_11553 Phosphotransferase
Ga0073283_103131 Glucose-6-Phosphate Isomerase
Ga0073283_10741 Phosphofructokinase
Ga0073283_11516 Phosphofructokinase
Ga0073283_11945 Phosphofructokinase
Ga0073283_12818 Phosphofructokinase
Ga0073283_10613 Fructose bisphosphate aldolase
Ga0073283_10765 Fructose bisphosphate aldolase
Ga0073283_10414 Triose Phosphate Isomerase
Ga0073283_10412 Glyceraldehyde-3-phosphate dehydrogenase
Ga0073283_10413 3-phosphoglycerate kinase
Ga0073283_10415 Phosphoglycerate mutase
Ga0073283_10416 Enolase
Ga0073283_11944 Pyruvate Kinase
Ga0073283_11648 Pyruvate-Ferredoxin Oxidoreductase
Ga0073283_11540 Pyruvate Formate Lyase
Ga0073283_1281 Pyruvate Formate Lyase
Ga0073283_1397 Pyruvate Formate Lyase
Ga0073283_102157 Lactate Dehydrogenase
Ga0073283_101140 Phosphotransacetylase
Ga0073283_13613 Phosphotransacetylase
Ga0073283_10936 Phosphotransacetylase²
Ga0073283_10940 Acetate Kinase³
Ga0073283_101139 Acetate Kinase
Ga0073283_10944 Aldehyde Dehydrogenase
Ga0073283_10941 Alcohol Dehydrogenase⁴
Ga0073283_11320 Alcohol Dehydrogenase¹
Ga0073283_11552 Alcohol Dehydrogenase*
Ga0073283_11934 Alcohol Dehydrogenase
Ga0073283_1491 Alcohol Dehydrogenase
Ga0073283_1513 Alcohol Dehydrogenase
Ga0073283_1238 Methylglyoxal Synthase
Ga0073283_104122 Glyoxalase
Ga0073283_10537 Methylglyoxal Reductase
Ga0073283_11338 Methylglyoxal Reductase
Ga0073283_12826 Methylglyoxal Reductase
Ga0073283_11442 Methylglyoxal Reductase
Ga0073283_11320b 1,2-Propanediol Dehydrogenase¹
Ga0073283_11552b 1,2-Propanediol Dehydrogenase*
Ga0073283_11728 FeFe Hydrogenase
Ga0073283_11749 Hydrogenase Maturation Factor
Ga0073283_12755 FeFe Hydrogenase
Ga0073283_1384 NADP-reducing Hydrogenase
Ga0073283_1385 NADP-reducing Hydrogenase
Ga0073283_1386 NADP-reducing Hydrogenase
Ga0073283_1485 Coenzyme F420-reducing Hydrogenase
Ga0073283_11368 Hydrogenase Maturation Factor
Ga0073283_11366 Hydrogenase System Regulator
Ga0073283_10954 Propanediol Dehydratase (small subunit)
Ga0073283_10955 Propanediol Dehydratase (medium subunit)
Ga0073283_10956 Propanediol Dehydratase (large subunit)
Ga0073283_10952 Diol Dehydratase Reactivation Enzymes
Ga0073283_10953 Diol Dehydratase Reactivation Enzymes
Ga0073283_10938 Propanediol Utilization Protein (PduV)
Ga0073283_10943 Cobalamin Reductase (PduS)
Ga0073283_10945 ATP:Cob(I)alamin Adenosyltransferase (PduO)
Ga0073283_10946 Cob(I)alamin Adenosyltransferase
Ga0073283_10986 Cob(I)alamin Adenosyltransferase⁵
Ga0073283_10948 Microcompartment Assembly Protein (PduM)
Ga0073283_10936b Phosphotransacetylase (PduL)²
Ga0073283_10940b Acetate Kinase (PduW)³
Ga0073283_10941b Alcohol Dehydrogenase (PduQ)⁴
Ga0073283_10944b Propionaldehyde Dehydrogenase (PduP)
Ga0073283_10949 Chaperone Protein
Ga0073283_10942 Carboxysome Shell Protein
Ga0073283_10947 Carboxysome Shell Protein
Ga0073283_10950 Carboxysome Shell Protein
Ga0073283_10951 Carboxysome Shell Protein
Ga0073283_10957 Carboxysome Shell Protein
Ga0073283_10958 Carboxysome Shell Protein
Ga0073283_10939 Carboxysome Shell Protein
Ga0073283_10970 L-threonine Kinase⁶
Ga0073283_10961 Glutamate Semialdehyde Aminomutase
Ga0073283_10962 Porphobilinogen Synthase
Ga0073283_10963 Uroporphyrinogen III Synthase
Ga0073283_10981 Precorrin-2 C20 Methyltransferase
Ga0073283_10975 Precorrin-3B C17 Methyltransferase
Ga0073283_10980 Precorrin-4 C11 Methyltransferase
Ga0073283_10973 Precorrin-5A Hydrolase
Ga0073283_10984 Precorrin-5B C1 Methyltransferase
Ga0073283_10974 Precorrin-6A Reductase
Ga0073283_10982 Precorrin-6B C15 Methyltransferase
Ga0073283_10983 Precorrin-7 C5 Methyltransferase
Ga0073283_10985 Precorrin-8 Methylmutase
Ga0073283_10972 Cobyrinic Acid A,C-diamide Synthase
Ga0073283_10986b Cob(I)alamin Adenosyltransferase⁵
Ga0073283_10987 Adenosylcobyric Acid Synthase
Ga0073283_10988 Adenosylcobyric Acid Synthase
Ga0073283_10970b L-threonine Kinase⁶
0.1 MPa
21 MPa
35 MPa
48 MPa

Supplemental Figure 1. Heatmap highlighting all proteins involved in carbon flux used to generate Figure 3. Each column represents a different pressure, proteins are clustered based on metabolic pathway (y-axis). Low Z-score values are represented by blues and shift to red as Z-scores increase. Purple represent similar protein concentrations across all pressure gradients. Grey boxes indicated proteins that were undetected. Black outlined boxes represent a significant difference ($p < 0.05$, student t-test) in protein abundance between atmospheric and high pressure conditions. No outline represents changes in protein abundance that were not statistically significant ($p > 0.05$, student t-test). Some proteins listed may play roles in multiple metabolic pathways listed, and matching proteins are denoted with a superscript. Protein names in red were used to make the arrow colors in Figure 3.

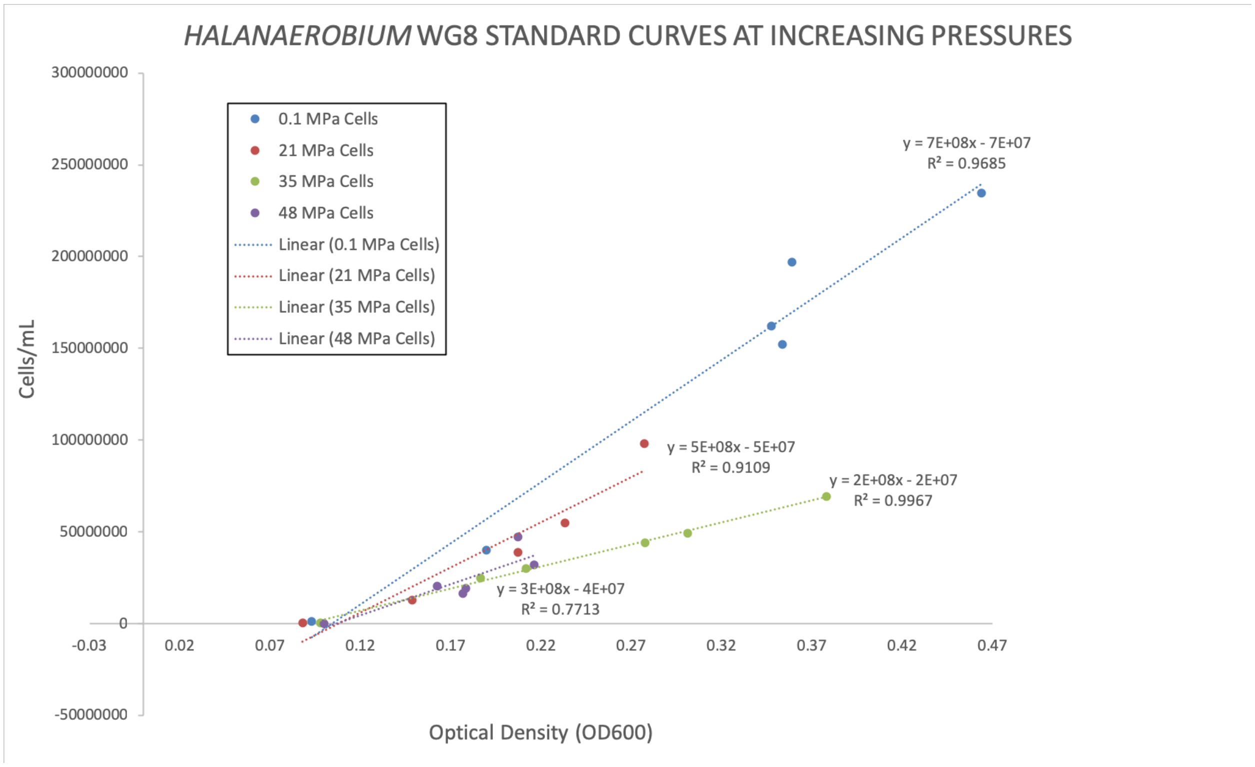

Supplemental Figure 2. Standard curves used to convert optical density measurements (OD) to cells/mL. There is one standard curve for each of the four pressures presented in this study (0.1, 21, 35, and 48 MPa denoted as ATM, 3000, 5000, and 7000 PSI respectively). Measured optical density is labeled on the x-axis with corresponding cells/mL on the y-axis.

Supplemental Table 1. Balanced oxidation/reduction (O/R) potentials of *H. congolense* WG8 growth at 0.1 and 35 MPa.

Supplemental Table 2. Shotgun proteomic results for *H. congolense* WG8 cultured at the four pressure conditions.

Supplemental Table 3. PLFA profiles for *H. congolense* WG8 at 0.1 and 48 MPa
